# Mutation of Gemin5 Causes Defective Hematopoietic Stem/Progenitor Cells Proliferation in Zebrafish Embryonic Hematopoiesis

**DOI:** 10.3389/fcell.2021.670654

**Published:** 2021-04-30

**Authors:** Xiaofen Liu, Wenjuan Zhang, Changbin Jing, Lei Gao, Cong Fu, Chunguang Ren, Yimei Hao, Mengye Cao, Ke Ma, Weijun Pan, Dantong Li

**Affiliations:** ^1^Shanghai Jiao Tong University School of Medicine, Shanghai, China; ^2^Shanghai Institute of Nutrition and Health, Chinese Academy of Sciences, Shanghai, China; ^3^Clinical Research and Translation Center, The First Affiliated Hospital of Fujian Medical University, Fujian, China

**Keywords:** Gemin5, Hematopoietic stem/progenitor cells, cell proliferation, zebrafish, definitive hematopoiesis, forward genetic screening, positional cloning

## Abstract

Fate determination and expansion of Hematopoietic Stem and Progenitor Cells (HSPCs) is tightly regulated on both transcriptional and post-transcriptional level. Although transcriptional regulation of HSPCs have achieved a lot of advances, its post-transcriptional regulation remains largely underexplored. The small size and high fecundity of zebrafish makes it extraordinarily suitable to explore novel genes playing key roles in definitive hematopoiesis by large-scale forward genetics screening. Here, we reported a novel zebrafish mutant line *gemin5*^*cas008*^ with a point mutation in *gemin5* gene obtained by ENU mutagenesis and genetic screening, causing an earlier stop codon next to the fifth WD repeat. Gemin5 is an RNA-binding protein with multifunction in post-transcriptional regulation, such as regulating the biogenesis of snRNPs, alternative splicing, stress response, and translation control. The mutants displayed specific deficiency in definitive hematopoiesis without obvious defects during primitive hematopoiesis. Further analysis showed the impaired definitive hematopoiesis was due to defective proliferation of HSPCs. Overall, our results indicate that Gemin5 performs an essential role in regulating HSPCs proliferation.

## Introduction

In vertebrates, embryonic development of blood system involves two waves of hematopoiesis: the primitive hematopoiesis and the definitive hematopoiesis. In definitive wave, the fate of Hematopoietic Stem and Progenitor Cells (HSPCs) is determined in the aorta-gonad-mesonephros (AGM) region, and then generating all blood lineages throughout the life (Galloway and Zon, 2003). Ontogeny from HSPCs fate determination to expansion and then terminal differentiation of types of blood cells requires an orchestrated regulation of transcriptional networks and signaling pathways, however, its underlying mechanism remains largely unknown.

Recently, zebrafish as a wonderful vertebrate model system, has become popular in hematopoiesis research. With high fecundity and small size, zebrafish extremely suits large-scale forward genetics screening (Gao et al., 2015). Taking the benefit of the external fertilization, we can observe and manipulate on zebrafish embryos as early as the one-cell stage. In addition, definitive hematopoiesis is highly conserved between zebrafish and mammals (Song et al., 2004; Gore et al., 2018). In zebrafish, HSPCs emerge from the ventral wall of the dorsal aorta, equals to the AGM in mammals, through endothelium to hematopoietic transition (Kissa and Herbomel, 2010). Then they migrate and colonize in the caudal hematopoietic tissue (CHT), which is similar to mammalian fetal liver, for HSPCs rapid expansion and the differentiation of erythroid and myeloid cells (Ciau-Uitz et al., 2014). By 4 days post-fertilization (dpf), the HSPCs migrate to the kidney marrow, analogous to the bone marrow of mammals (Murayama et al., 2006). Although the main hematopoietic sites are different in zebrafish and mammals, both of them share the same major blood cell types arising from common hematopoietic lineages (Traver et al., 2003), which means findings of blood development in zebrafish could be translated to mammalian systems.

Normal function of HSPCs is sustained through an orchestrated regulation at transcriptional and post-transcriptional level. Major advances have been achieved in the understanding of transcriptional regulation of HSPCs; however, it remains underexplored for the post-transcriptional regulation. RNA-binding proteins (RBPs) take control of post-transcriptional regulation in hematopoiesis through modulating RNA properties including RNA splicing, localization, degradation, or translation (de Rooij et al., 2019). It has been reported RBPs have essential roles in regulating cell fate determination and cell proliferation in stem cell biology (Grech and von Lindern, 2012; de Rooij et al., 2019). Gemin5, an RBP with multidomain, consists of the *N*-terminal tryptophan-aspartic acid (WD) repeat domains, a dimerization domain in the middle, and a non-canonical RNA-binding domain in the *C*-terminal region (Martinez-Salas et al., 2020). Its WD repeat motifs recognize the small nuclear RNAs (snRNAs), allowing assembly of the survival of motor neurons (SMN) complex into small nuclear ribonucleoproteins (snRNPs; Pineiro et al., 2015; Martinez-Salas et al., 2020). Structure analyses show the dimerization module of Gemin5 acts a fundamental part in its architecture and essential for its activity (Martinez-Salas et al., 2020). The non-canonical RNA-binding domain allows Gemin5 to interact with its mRNA targets and play a key role in translation. Research on Gemin5 is emerging but far from enough to understand the consequence of its multifunction.

Since ENU-mutagenesis combined with forward genetic screening is a powerful approach to unbiasedly uncover novel genetic and molecular pathways, we conducted it in zebrafish model organism to dissect the sophisticated mechanisms of definitive hematopoiesis (Gao et al., 2015; Jia et al., 2015). We first generated mutants by mutagen ethylnitrosourea (ENU). Then we did whole mount *in situ* hybridization (WISH) on the embryos of F2 families with *myb* probe, a crucial marker and transcription factor of HSPCs. We obtained a new mutant *cas008* showing severe hematopoietic defects. Then we accomplished positional cloning and located the mutation in gene *gemin5*. Furthermore, we found abnormal definitive hematopoiesis was due to defective proliferation of HSPCs. To sum up, we reported a zebrafish mutant family carrying Gemin5 point mutation with severe definitive hematopoiesis defects due to HSPCs proliferation failure.

## Materials and Methods

### Zebrafish Husbandry

The zebrafish facility and studies were approved by the Animal Research Advisory Committee of Shanghai Institute of Nutrition and Health, CAS. Zebrafish were kept in accordance with the guidelines of the Institutional Animal Care and Use Committee (Li et al., 2018).

The transgenic zebrafish line Tg(*myb*: eGFP; North et al., 2007) was described previously. For the forward genetics screen, Wild-type (WT) TU zebrafish line was treated with ENU, Sigma (Gao et al., 2015; Jia et al., 2015). For positional cloning, we first outcrossed heterozygous *mutant*^*cas008*^ in Tu background with wild-type in polymorphic WIK background and then mapped the *mutant*^*cas008*^ allele in Tu background as described previously (Bahary et al., 2004).

### Mapping and Identification of Mutation in Zebrafish *cas008* Mutant

Based on the *myb* expression in zebrafish embryos at 5 dpf, the *mutant*^*cas008*^ line was identified among numerous ENU-mutagenized F2 families. We carried on the ENU screen and positional cloning as mentioned before (Bahary et al., 2004; Gao et al., 2015; Jia et al., 2015). By bulk segregation analysis with sequence length polymorphism (SSLP) markers, the mutation was first mapped to chromosome 21. To narrow down the genetic interval, fine mapping was performed and identified the mutation flanked by two SSLP markers, zK189O20 and zC153M12. Then, we cloned the cDNAs of candidate genes in the range and sequenced from siblings and mutants separately. Finally, we sequenced genomic DNA of individual mutant embryos to confirm the putative mutation.

### Plasmid Construction and Microinjection

We cloned the zebrafish Gemin5 (accession number: ZDB-GENE-031112-9) and inserted it into the Tol2 backbone between the promoter UAS and p2A (primer F: atgcacgaaagacatctgcc; primer R: gtgtatgagtctcctcacag) (Schematic diagram in Figure 2C). For transient transgene, Tol2 transposase mRNA (40 pg) and the Gemin5 construct or Tol2 vector (40 pg) were microinjected into one-cell-stage embryos (Suster et al., 2009).

### WISH, FISH, TUNEL Assay and Immunofluorescence Staining

The *myb*, *ae1-globin*, *lyz*, *mpx*, *rag1*, *gata1*, *pu.1*, and *kdrl* probe were transcribed by polymerase T3 or T7 (Ambion) with Digoxigenin RNA Labeling Mix (Roche) *in vitro* (Li et al., 2018). WISH was carried out as described before using NBT/BCIP (Sigma) as substrates. To detect HSPCs proliferation, we performed fluorescence whole mount *in situ* hybridization (FISH) and pH3 immunofluorescence double-staining as described previously (Gao et al., 2015; Jia et al., 2015). We first stained embryos with cy3 (TSA system, Perkin Elmer), and used anti-pH3 (ser10) antibody (1:500; Santa Cruz) for pH3 immunofluorescence staining, and then imaged by Olympus FV1000 Fluoview scanning confocal microscope. With *in situ* Cell Death Detection Kit and TMR Red Kit (Roche), TUNEL assay was conducted according to manufacturer’s instruction. Embryos in Tg(*myb*:eGFP) background were fixed with 4% PFA. After pretreatment, the embryos were stained at 37°C for 2 h using the TUNEL Kit (100 μl, enzyme: labeling solution = 1:9). Next, the immunostaining of eGFP was performed with incubation of anti-GFP (Rabbit, 1:500, Invitrogen) and secondary antibody goat-Alexa Fluore488-conjugated anti-rabbit (1:500, Invitrogen). Then, the embryos were imaged by confocal microscope Olympus FV1000 Fluoview scanning.

### Imaging

Images of zebrafish immunofluorescence staining (Figures 3A,B and Supplementary Figure 3A) were taken by Olympus FV1000 Fluoview scanning confocal microscope. 1% low-melt agarose was used to mount the embryos. These confocal images were captured by UPLSAPO 20X objective. Scale bars, 50 μm.

### Statistical Analysis

Data were analyzed with the two-tailed Student’s *t*-test using the software Graphpad Prism 8. Error values in the plots were calculated by standard error of the mean (SEM). In this study, all data were repeated for at least twice.

## Results

### Obtained Zebrafish *Mutant^*cas008*^* Line in a Large-Scale Forward Genetics Screen

**FIGURE 1:**
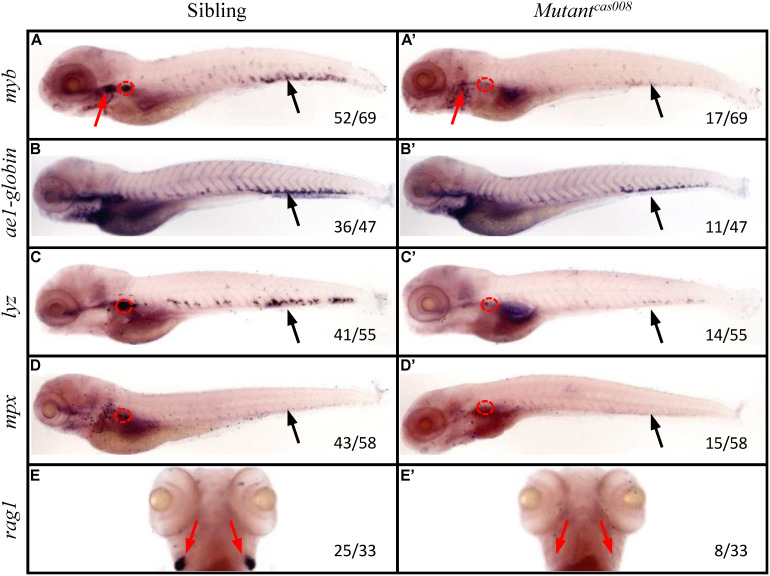
The *mutant*^*cas008*^ line showed defective definitive hematopoiesis. WISH analysis of *myb* **(A,A′)**, *ae1-globin* **(B,B′)**, *lyz* **(C,C′)**, *mpx* **(D,D′)**, and *rag1* **(E,E′)** expression in sibling **(A–E)** and *mutant*^*cas008*^ **(A′–E′)** embryos at 5 dpf. The penetrance of the indicated phenotype is shown in the bottom right of each panel. Black arrows indicate the position of CHT, while red arrows and circles show the position of thymus and kidney, respectively.

To explore novel genes which played essential roles in definitive hematopoiesis, we used zebrafish model organism to perform a large-scale forward genetics screen (Gao et al., 2015; Jia et al., 2015). First of all, we generated mutants by crossing zebrafish previously treated with ENU mutagen. Then we tested *myb* expression by WISH on the embryos of F2 families. As a key transcription factor and marker of HSPCs, *myb* was expressed in all hematopoietic tissues of sibling embryos at 5 dpf, including CHT, thymus and kidney tested by WISH (Figure 1A). While *mutant*^*cas008*^ displayed dramatically decreased *myb* expression in CHT, thymus and kidney suggesting defective definitive hematopoiesis (Figure 1A′). The cell marker of downstream hematopoietic lineages, including *ae1-globin* (erythrocyte marker), *lyz* (macrophage marker), *mpx* (granulocyte marker), and *rag1* (lymphocyte marker) were also examined. Comparing with siblings, *mutant*^*cas008*^ expressed decreased *ae1-globin* and barely expressed *lyz*, *mpx*, and *rag1* (Figures 1B–E,B′–E′). All these results showed definitive HSPCs in the CHT are severely disrupted and downstream differentiation of HSPCs were blocked in *mutant*^*cas008*^ embryos.

Furthermore, we tested the primitive hematopoiesis in *mutant*^*cas008*^ by WISH. The results showed the expression of primitive hematopoietic cell markers, including *gata1* (erythrocyte progenitor marker) and *pu.1* (myeloid progenitor marker; Supplementary Figures 1A,B,A′,B′) were identical between siblings and *mutant*^*cas008*^ at 22 hpf. As mentioned, in the developing dorsal aorta HSPCs emerge from endothelial cells during 33–54 hpf (Kissa and Herbomel, 2010), thus vascular morphogenesis is essential for HSPCs initiation and maintenance (Burns et al., 2009). The expression of the pan-endothelial cell marker *kdrl* at 26 hpf before HSPCs emergence was examined and it was intact in *mutant*^*cas008*^ as well (Supplementary Figures 1C,C′). We also examined the expression of *runx1* at 36 hpf, required for HSPCs emergence and showed no defects in *mutant*^*cas008*^ comparing with sibling embryos (Supplementary Figures 1D,D′). Taken together, we obtained *mutant*^*cas008*^ displayed specific deficiency in definitive hematopoiesis without obvious defects during primitive hematopoiesis, vasculogenesis and HSPCs emergence.

### Positional Cloning

**FIGURE 2:**
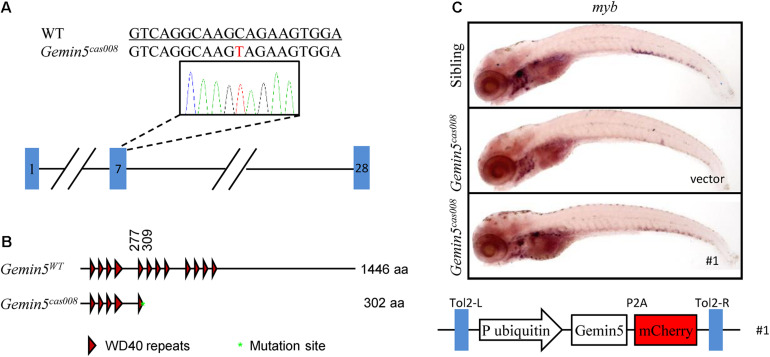
The mutation was mapped to a point mutation in gene *gemin5*. **(A)** The alignment of WT (underlined) and NC_007132.7: g.36404684 C > T (*gemin5*^008^) sequences are listed. The mutation by ENU is indicated in red (a C to T nonsense mutation causes an immediate stop codon in *gemin5* in *mutant*^*cas008*^). **(B)** According to the stop codon in the genome, SMART software was used to predict the structure of Gemin5^*WT*^ and XP_001339880.4:p.Gln303_His1440del (Gemin5^008^) presumed protein. The molecular sizes of the presumed protein are indicated on the right. **(C)** WISH analysis with *myb* probe in the CHT region (at 6 dpf) of sibling and *gemin5^008^* with transient transgenesis of vector (only plasmid backbone) or ubi: gemin5 (#1, The construction of the plasmid is shown below).

We carried out positional cloning to elucidate the mechanism underlying the hematopoietic failure in *mutant*^*cas008*^. According to the previously reported procedure (Gao et al., 2015; Jia et al., 2015), by bulk segregation analysis (BSA) the mutation was first mapped to chromosome 21. Then a high-resolution mapping approach was performed and it identified the mutation was flanked by two linked SSLP markers, zK189O20 and zC153M12. There were four candidate genes in the flanked region (Supplementary Figure 2A). Among them, we identified a C to T nonsense mutation causing an immediate stop codon in *gemin5* in *mutant*^*cas008*^ by sequencing cDNA of the four genes (NC_007132.7: g.36404684 C > T, Figure 2A). Next, the result was verified by genomic sequencing. The earlier stop codon caused by this mutation was next to the end of the fifth WD repeats domain of Gemin5 protein (XP_001339880.4:p. Gln303_His1440del, Figure 2B). Hence, we changed the name of *mutant*^*cas008*^ into NC_007132.7: g.36404684 C > T, *gemin5*^*cas008*^ for short. To further confirm our results, we performed a rescue experiment by employing a Tol2 transposase-mediated transgenic approach to express wild-type (WT) g*emin5* gene in *gemin5*^*cas008*^ embryos. In the construction of transgenic plasmid, the ubiquitin promoter drove ubiquitous expression of wildtype *gemin5*, followed by P2A peptide-mCherry fusion protein to indicate transgenic efficiency (P2A peptide allows self-cleavage to avoid influencing Gemin5 function). This plasmid element was flanked by Tol2 arms (Figure 2C). After co-injection of the plasmid and Tol2 transposase mRNA into one-cell stage *gemin5*^*cas008*^ embryos, ubiquitous expression of wild-type *gemin5* could rescue *gemin5*^*cas008*^ phenotype to a large extend at 6 dpf (Figure 2C). Taken together, the nonsense mutation in *gemin5* caused the defective hematopoietic phenotypes in *gemin5*^*cas008*^.

Gemin5, as an RBP with multidomain consists of the *N*-terminal WD repeat domains, a dimerization domain in the middle, and a non-canonical RNA-binding domain in the *C*-terminal region (Martinez-Salas et al., 2020). Its WD repeat motifs recognize the snRNAs, allowing assembly of the SMN complex into snRNPs (Pineiro et al., 2015; Martinez-Salas et al., 2020). Structure analyses show the dimerization module of Gemin5 is fundamental for its architecture and plays an essential role in its activity (Martinez-Salas et al., 2020). The non-canonical RNA-binding domain allows Gemin5 to interact with its mRNA targets and play a key role in translation. Research on Gemin5 is emerging but far from enough to understand the consequence of its multifunction, including its canonical role in the biogenesis of snRNPs, and its control on alternative splicing and translation, etc. (Pineiro et al., 2015).

### HSPCs Failed in Proliferation in *Gemin5*^*cas008*^

**FIGURE 3:**
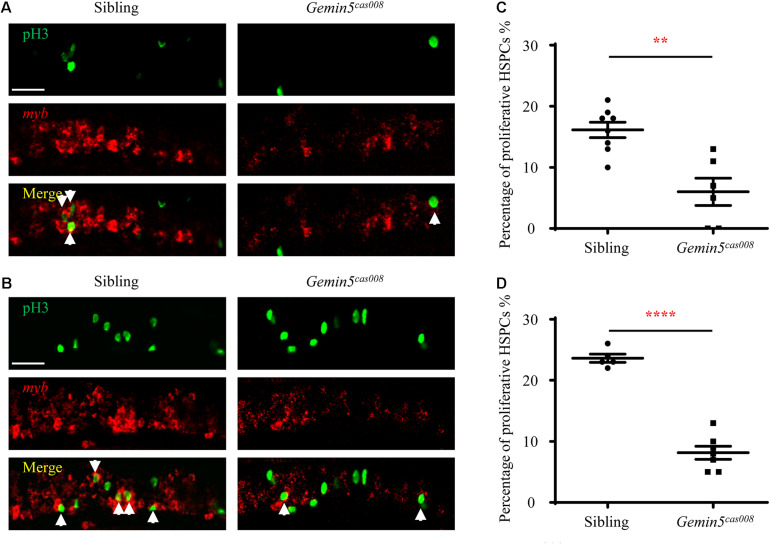
Decreased HSPCs proliferation in mutant *gemin5*^*cas008*^. **(A,B)** Representative confocal images of double staining of pH3 protein (green) and *myb* RNA (red) in the CHT at 4 dpf **(A)** and 5 dpf **(B)**. Arrows point at proliferative HSPCs. Scale bars, 50 μm. **(C,D)** Percentage of proliferative HSPCs (pH3+myb+)/ total HSPCs (myb+) in sibling and *gemin5*^*cas008*^ embryos at 4 dpf **(C)** and 5 dpf **(D)**. **(C)** *t* = 4.202, df = 12, and *P* = 0.0012. **(D)** *t* = 10.96, df = 10, and *P* < 0.0001.

To elucidate the reason of HSPCs abrogation, we first examined HSPCs proliferation during hematopoietic development by combination of fluorescence *in situ* hybridization (FISH) and phospho-histone 3 (pH3) immunostaining. We calculated the percentage of proliferative HSPCs (pH3 + *myb*+) in total HSPCs (*myb*+). At both 4 and 5 dpf, the percentage of proliferative HSPCs (pH3^+^ *myb*^+^) in the CHT in *gemin5*^*cas008*^ was significantly decreased (Figures 3A–D, 4 dpf *P* = 0.0012, and 5 dpf *P* < 0.0001). Next, we examined the apoptosis of HSPCs by terminal-transferase dUTP Nick End Labeling (TUNEL) assays in transgenic background Tg(*myb*:eGFP) and calculated the percentage of apoptotic HSPCs (TUNEL+ GFP+) in total HSPCs (GFP+). Apoptotic signals of HSPCs in the CHT region had no significant difference between sibling and *gemin5*^*cas008*^ at 4 dpf (Supplementary Figures 3A,B, *P* = 0.1430), which indicated there was no excessive HSPCs apoptosis in mutant *gemin5*^*cas008*^. In brief, impaired definitive hematopoiesis in *gemin5*^*cas008*^ was due to defective proliferation of HSPCs.

In brief, we obtained a zebrafish mutant family with Gemin5 point mutation. Mutant *gemin5*^*cas008*^ showed no obvious defects during primitive hematopoiesis or vasculogenesis. However, the reductive HSPCs proliferation caused severe definitive hematopoiesis failure in the *gemin5*^*cas008*^.

## Discussion

The basis of stem cells’ self-maintenance and differentiation could be described as genetically programmed and integrated circuits (Yuan and Muljo, 2013). In the hematopoietic system, major advances have been achieved in elucidating the regulatory networks which orchestrate gene expression programs and direct cell-fate determinations. Among them, much work has focused on elucidating signaling pathways and transcriptional programs. However, the post-transcriptional regulation of HSPCs is far from well-explored. This “RNA centric” level of regulation precisely and quickly tunes protein expression by regulating splicing, localization, degradation, or translation of mRNA (de Rooij et al., 2019). As one of the key players, RBPs perform their function by interaction with existent transcriptional networks in the cell. They interact with their target mRNAs which are based on RNA sequence, strongly influencing their downstream processing. It has been reported different changes of RBPs will cause the disruption of hematopoietic homeostasis (Yuan and Muljo, 2013). For instance, the RBP Rbm15 regulates splicing of Mpl-TR, the truncated isoform of the c-Mpl receptor, which affects thrombopoietin signaling and subsequently influences HSCs quiescence and proliferation (Xiao et al., 2015). The oncogenic fusion gene BCR-ABL markedly increased the expression of the RBPs La/SSB, multiple hnRNPs and TLS/FUS and interfered mRNA processing, nuclear-cytoplasmic export and translation, resulting in the unbalance of HSCs proliferation and differentiation (Perrotti and Calabretta, 2002).

In this study, we obtained a zebrafish mutant family with Gemin5 point mutation through a large-scale forward genetics screen. Gemin5 is an RBP described as a peripheral component of the SMN complex, first identified in 2002 (Gubitz et al., 2002). In eukaryotic cells, the SMN complex acts as an assemblyosome, mediating the biogenesis and function of spliceosomal snRNPs. It is intensive in nuclear Gems (Gemini of Cajal bodies), though it is dispersed throughout the cytoplasm. The SMN complex is composed of nine members, including SMN, Gemins2–8 and the unr-interacting protein (unrip) in human (Martinez-Salas et al., 2020). Gemin5 comprises WD repeat domains at the *N*-terminal, recognizing the snRNAs, delivering them to the SMN complex and allowing assembly of the SMN complex and snRNAs into snRNPs in the cytoplasm. Gemin5 is predominantly distributed in the cell cytoplasm (Gubitz et al., 2002). However, a large fraction of Gemin5 is found outside of the SMN complex (Battle et al., 2007), which suggests there may be additional functions of Gemin5. In accordance with this, it has been detected Gemin5 and the SMN complex colocalize in nuclear gems, rather than in nuclear Cajal bodies. Gemin5 may participate in tumor cell motility through the alternative splicing process. For instance, analyze the global mRNA splicing profile of the MDA-MB-435 tumor cell line at metastatic state or suppression state, showing a distinct splicing profile between different cellular states in dependence on Gemin5 (Lee et al., 2008, 2009). In addition, it has been reported that Gemin5 may interact with signal recognition particle or ribosome, and regulate the translation or *trans*-splicing of several mRNAs. More recently, structural analyses have unveiled there is a robust dimerization module in Gemin5, helping understand the functional role of the middle region (Moreno-Morcillo et al., 2020). The dimerization module of Gemin5 is fundamental in its architecture and essential for its activity. At the *C*-terminal region, it has a non-canonical RNA-binding site (RBS) with two parts named as RBS1 and RBS2, not only determining the interaction with IRES (viral internal ribosome entry site) of the FMDV (foot-and-mouth disease virus), but also harboring the capacity to downregulate translation (Fernandez-Chamorro et al., 2014). In sum, Gemin5 plays an essential role in the snRNPs biogenesis. Moreover, with the analysis of its structure, its function involved in alternative splicing, stress response and translation control has been uncovered. Although genetic evidence shown defective Gemin5 might link to neuron degeneration, its role in other tissues/organs remains unknown.

In mutant *gemin5*^*cas008*^, we identified a C to T nonsense mutation causing an earlier stop codon next to the end of the fifth WD repeats domain of Gemin5 protein (Figures 2A,B). Mutant *gemin5*^*cas008*^ had intact primitive hematopoiesis, while showed specific deficiency in definitive hematopoiesis. FISH and pH3 immunostaining results showed the percentage of proliferative HSPCs dramatically decreased in *gemin5*^*cas008*^ (Figures 3A–D). However, the percentage of apoptotic HSPCs had no significant difference (Supplementary Figures 3A,B). In accordance, inhibiting p53 dependent apoptosis pathway by p53 MO or bcl2 mRNA injection at one-cell stage could not rescue the phenotype of *gemin5*^*cas008*^ (similar experiments could rescue another mutant due to increased apoptosis in Gao et al., 2015). So, we concluded that impaired definitive hematopoiesis in *gemin5*^*cas008*^ was due to defective proliferation of HSPCs. It has been reported that in human cells, Gemin5 interact with snRNAs through the WD repeat domain. Particularly, RNA-mediated radical probing and mass spectrometry results showed the fifth WD repeat contributes to the interaction (Lau et al., 2009). In addition, the establishment of the interaction depends on the structural integrity of this domain, for even a point mutation at the W286 residue (human) would disrupt the interaction shown by mutational experiment. This residue in zebrafish Gemin5 orthologs (W270) is conserved and might be preserved in mutant *gemin5*^*cas008*^. Considering the intact primitive hematopoiesis and specific deficient definitive hematopoiesis, we speculate the truncated Gemin5 protein can still recognize snRNAs through its residual domain for there is no large-scale transcriptional splicing disorder in the cell. However, the efficiency of the interaction may be reduced, and its target may even be biased toward certain snRNAs, eventually leading to the appearance of specific transcription and splicing disorders, thereby targeting the hematopoietic process.

In addition, Gemin5 has cap-binding capacity in the WD repeat domains and may influence translation initiation in a cap-dependent manner (Pineiro et al., 2015). W286 and F338 (human), as the putative cap-interacting residues, are identified by combination of the protein homology/analogy recognition engine (PHYRE) algorithm and site-directed mutagenesis. These sites are conserved in zebrafish (W270 and F322), and W270 might be preserved while F322 was not in the mutant. As mentioned, truncated Gemin5 protein lacked C-terminal which interacts with IRES and regulates transition. It’s reasonable to believe the regulation of Gemin5 in translation initiation was partially preserved in the mutants. The defective Gemin5 might still maintain various basic functions to let the mutants survive early development without obvious defects, however, the biological processes during definitive hematopoiesis were beyond the ability of truncated Gemin5 and drove abnormal development. Based on reported researches about the regulation of Gemin5 on the ASK1-JNK1 signaling pathway (Kim et al., 2007) and its cooperation with nuclear receptor to transmit ecdysone signaling (Gates et al., 2004), we speculate some relatively sensitive signal pathways of HSPCs or the transcriptional regulatory network required for proliferation depending on Gemin5 were specifically disrupted in mutant *gemin5*^*cas008*^.

Questions remain open regarding how Gemin5 regulates hematopoiesis. First of all, the location and the protein level of the mutant Gemin5 protein need to be determined. Secondly, HSPC-specific re-expression of WT Gemin5 in *gemin5*^*cas008*^ need to be carried out to elucidate its requirement in a stem cell or niche cell manner. Lack of zebrafish Gemin5 antibody and lack of efficient HSPC-specific promoter for transgenesis need be overcome in future study. Thirdly, the pathogenic mechanism downstream of the mutated Gemin5 needs to be elucidated. It needs to be described which signal pathways of HSPCs or which transcriptional regulatory network required for proliferation depending on Gemin5 are essential. It will also be interesting to figure out the function of diverse regions of Gemin5 in the regulation of HSPCs. At last, it is worth identifying whether there are human hematopoietic diseases caused by Gemin5 mutations and what extent of Gemin5 involved in human diseases in blood system. It is very likely that the extend of other components in the SMN complex, as well as other RBPs, need be studied in hematopoiesis.

## Data Availability Statement

The original contributions presented in the study are included in the article/Supplementary Material, further inquiries can be directed to the corresponding author/s.

## Ethics Statement

The animal study was reviewed and approved by the Animal Research Advisory Committee of Shanghai Institute of Nutrition and Health, CAS.

## Author Contributions

XL, KM, WP, and DL designed the experiments. XL and KM carried on the experiments and analyzed data. CJ, LG, CF, and CR assisted with ENU screening and positional cloning. WZ, YH, and MC assisted with imaging with Olympus FV1000 Fluoview scanning confocal microscope. All the authors provided ideas and discussions throughout the project. WP and DL wrote the manuscript. KM, WP, and DL supervised the project.

## Conflict of Interest

The authors declare that the research was conducted in the absence of any commercial or financial relationships that could be construed as a potential conflict of interest.
