# Corrigendum: Mutation of Gemin5 causes defective hematopoietic stem/progenitor cells proliferation in zebrafish embryonic hematopoiesis

**DOI:** 10.3389/fcell.2024.1407675

**Published:** 2024-04-09

**Authors:** Xiaofen Liu, Wenjuan Zhang, Changbin Jing, Lei Gao, Cong Fu, Chunguang Ren, Yimei Hao, Mengye Cao, Ke Ma, Weijun Pan, Dantong Li

**Affiliations:** ^1^ Shanghai Jiao Tong University School of Medicine, Shanghai, China; ^2^ Shanghai Institute of Nutrition and Health, Chinese Academy of Sciences, Shanghai, China; ^3^ Clinical Research and Translation Center, The First Affiliated Hospital of Fujian Medical University, Fujian, China

**Keywords:** Gemin5, Hematopoietic stem/progenitor cells, cell proliferation, zebrafish, definitive hematopoiesis, forward genetic screening, positional cloning

In the published article, there were two errors in the **Funding** statement. Firstly, we inadvertently failed to include a crucial source of funding that played a significant role in supporting the research within the original **Funding** statement. Secondly, the grant number provided for The National Science Fund for Distinguished Young Scholars was incorrect. The original Funding statement was written as “The National Science Fund for Distinguished Young Scholars (Grant No. E034J11381), China National Postdoctoral Program for Innovative Talents (Grant No. BX20200347), and Shanghai Post-doctoral Excellence Program (Grant No. 2020505).” The correct **Funding** statement appears below.

