# Supplementary figures and images for: Mutation of Gemin5 Causes Defective Hematopoietic Stem/Progenitor Cells Proliferation in Zebrafish Embryonic Hematopoiesis
Source: Front Cell Dev Biol. 2021 Apr 30;9:670654. doi: 10.3389/fcell.2021.670654 (PMC8120239; doi:10.3389/fcell.2021.670654)

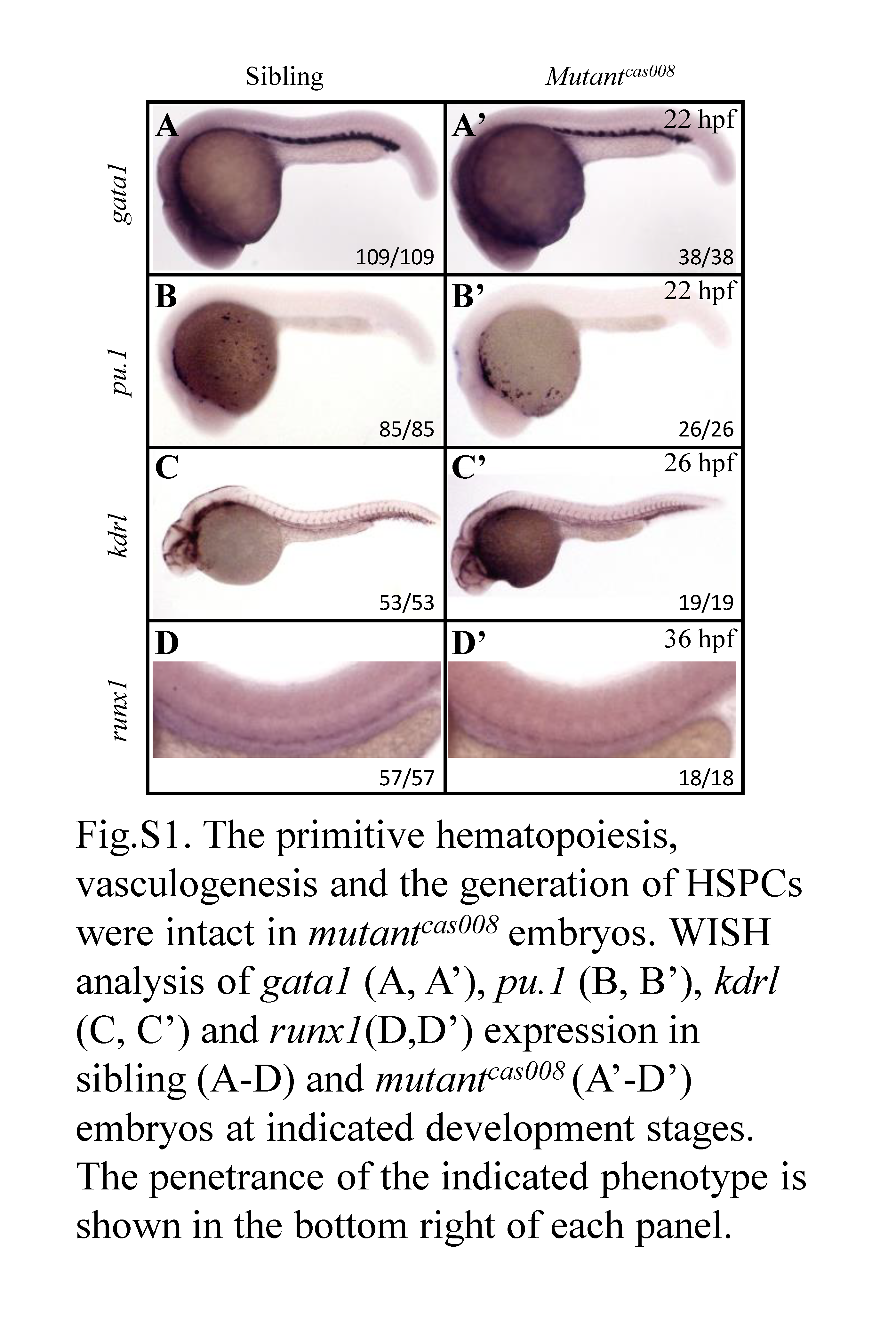

Supplement: Supplementary file 1 [file Image_1.TIF]

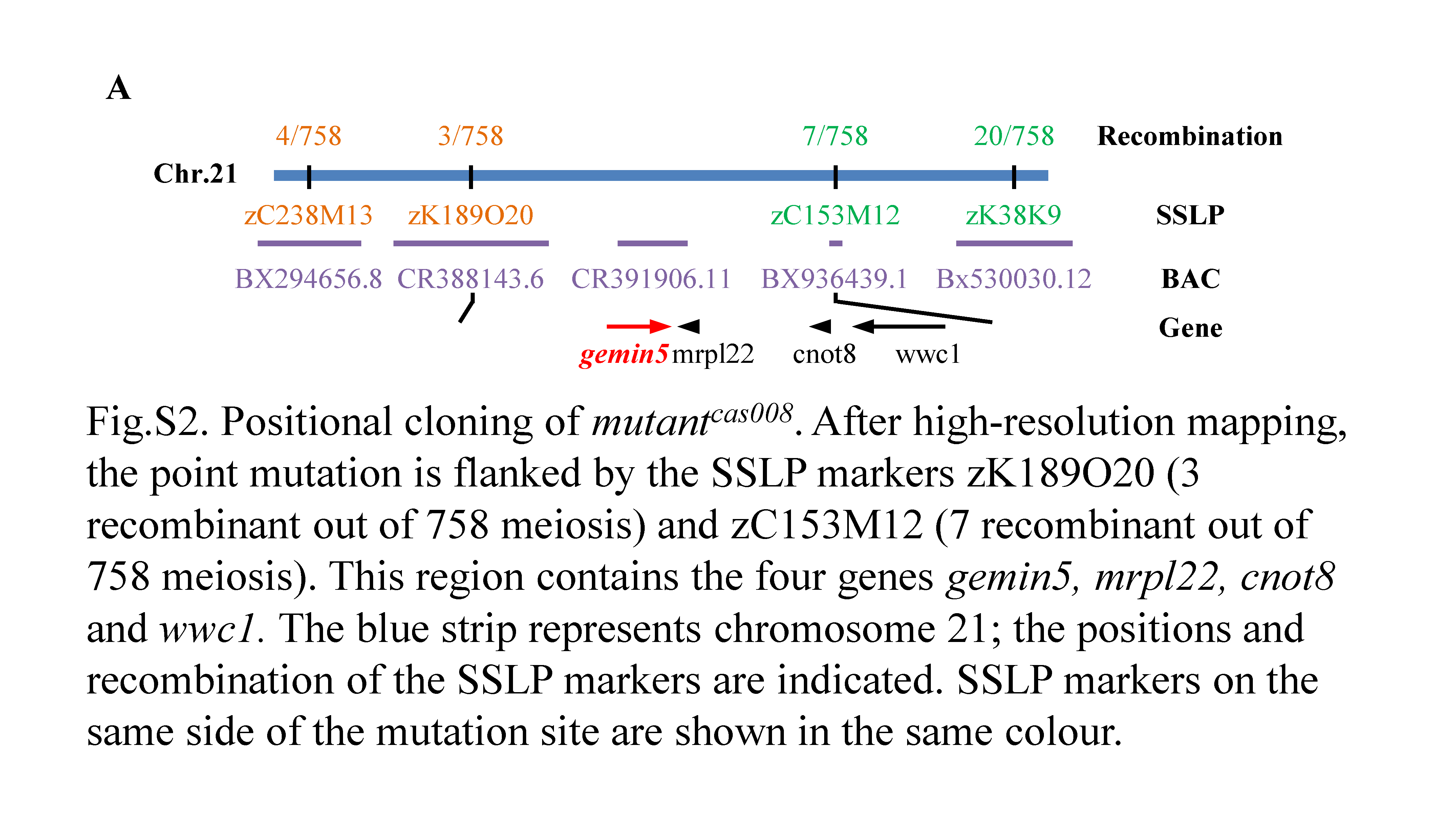

Supplement: Supplementary file 2 [file Image_2.TIF]

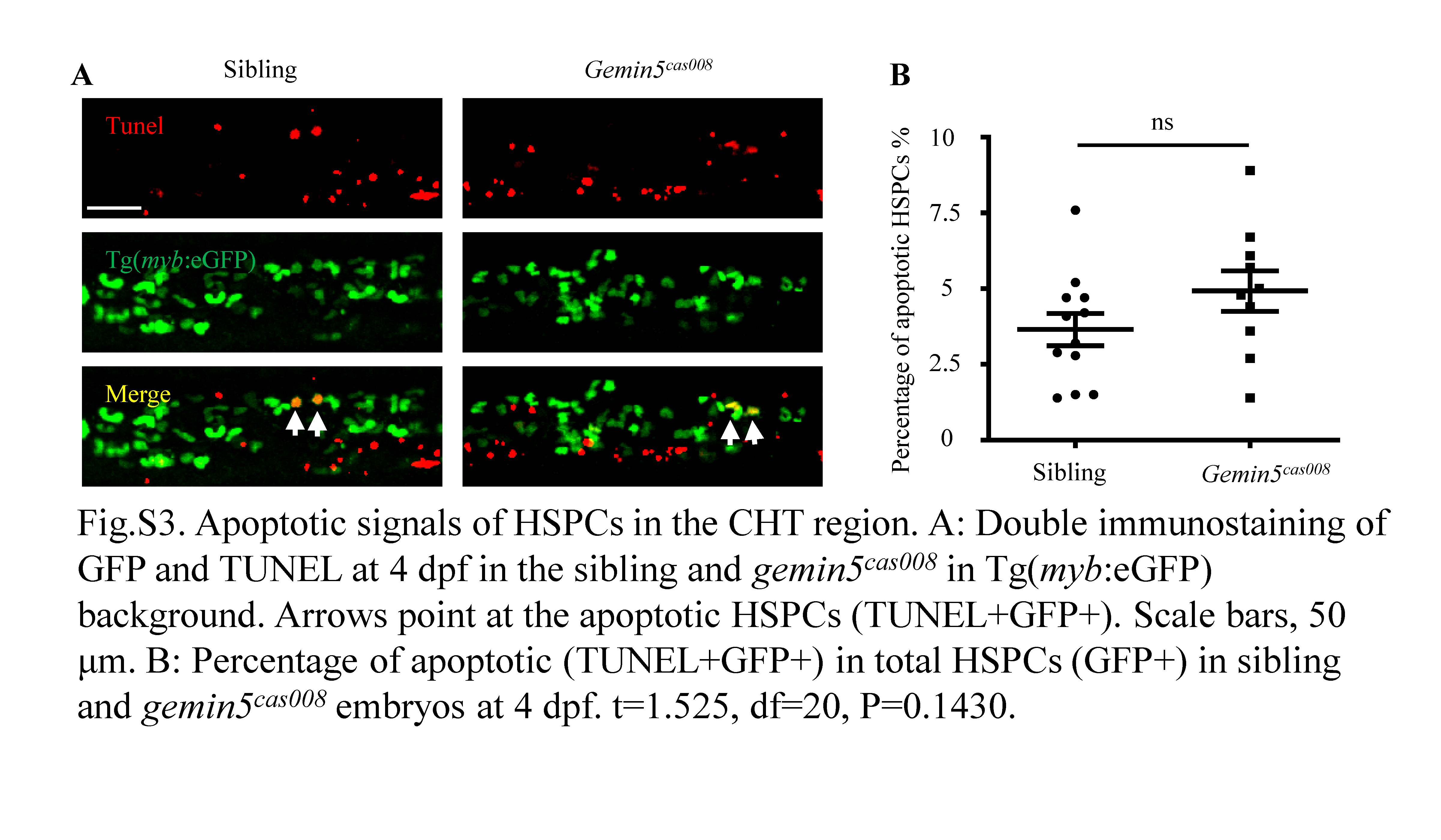

Supplement: Supplementary file 3 [file Image_3.TIF]
